# Factors Influencing the Detection of Spatially-Varying Surface Gloss

**DOI:** 10.1177/2041669519866843

**Published:** 2019-09-06

**Authors:** Gunnar Wendt, Franz Faul

**Affiliations:** Christian-Albrechts-Universität zu Kiel, Institut für Psychologie, Kiel, Germany

**Keywords:** 3D perception, gloss perception, object recognition, surfaces/materials

## Abstract

In this study, we investigate the ability of human observers to detect spatial inhomogeneities in the glossiness of a surface and how the performance in this task depends on several context factors. We used computer-generated stimuli showing a single object in three-dimensional space whose surface was split into two spatial areas with different microscale smoothness. The context factors were the kind of illumination, the object’s shape, the availability of motion information, the degree of edge blurring, the spatial proportions between the two areas of different smoothness, and the general smoothness level. Detection thresholds were determined using a two-alternative forced choice (2AFC) task implemented in a double random staircase procedure, where the subjects had to indicate for each stimulus whether or not the surface appears to have a spatially uniform material. We found evidence that two different cues are used for this task: luminance differences and differences in highlight properties between areas of different microscale smoothness. While the visual system seems to be highly sensitive in detecting gloss differences based on luminance contrast information, detection thresholds were considerably higher when the judgment was mainly based on differences in highlight features, such as their size, intensity, and sharpness.

## Introduction

Humans are able to recognize the material of an object solely on the basis of visual information (Adelson, 2001; Fleming, 2014), an ability that has been found to be fairly accurate and quick (Sharan, Rosenholtz, & Adelson, 2014; Wiebel, Valsecchi, & Gegenfurtner, 2013) and that seems to be established during early childhood (Balas, 2017). A common approach assumes that in order to determine the material of an object, a number of surface properties such as the diffuse color or texture, transparency, or glossiness would have to be estimated by the visual system (Fleming, Wiebel, & Gegenfurtner, 2013). Each material can then be represented by a specific combination of such surface properties (Fleming, 2017).

However, glossiness is not necessarily a constant property of a surface but can be subject to temporal changes or spatial inhomogeneities. It has been shown, for example, that the glossiness of several foods, such as carrots, strawberries, or fish, is negatively correlated with the degree of decomposition and that this information is used by human observers to judge their freshness (Murakoshi, Masuda, Utsumi, Tsubota, & Wada, 2013; Péneau, Brockhoff, Escher, & Nuessli, 2007). This demonstrates that temporal changes in the glossiness of an object can provide useful information about the current state of its material (although one may argue whether these are different states of the same material, for example, “carrot material,” or whether fresh and old carrots should be considered as different materials).

**Figure 1.**
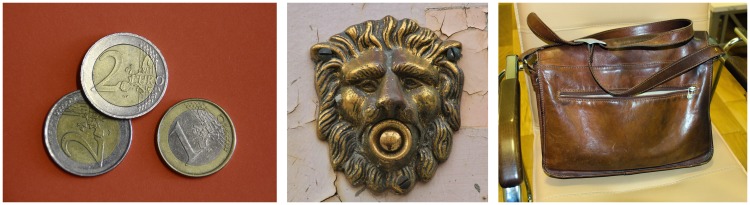
The occurrence of spatially-varying reflection characteristics on a surface can be due to several causes: Many objects are made of different materials, such as the Euro coins in the left image which consist of two parts with different metal alloys. Some surfaces are partly covered with layers of impurities, such as patina (center image). Other materials, like leather (right image), are comparatively sensitive to mechanical influences and can be easily roughened or polished which may locally affect their light reflecting behavior. (All images were taken from pixabay.com.)

One and the same object, especially when it is a man-made object or an object of utility, may have surface areas that differ in the degree of gloss (Figure 1). The most obvious cases are objects that are composed of different materials or whose surfaces have some local impurities, for instance, when they are covered with patina. Objects can also show more or less severe signs of wear or corrosion which could locally affect the roughness of their surface and therefore the way the incoming light is reflected. For example, objects with leather surfaces, such as boots, bags, wallets, or sitting furniture, can appear matte in some areas and shiny in other areas when they were accidentally roughened or polished in a spatially nonuniform manner. In addition to such mechanical influences, also chemical processes can locally change the gloss properties of a surface (see, e.g., Ged, Obein, Silvestri, Le Rohellec, & Viénot, 2010), where in some cases not only the microscale structure is changed but the material itself, for example, when iron turns into rust. Many of these local changes in the degree of gloss are also accompanied by changes in the diffuse color or the texture of the surface. In this study, however, we will focus on the question, to what extent the visual system is able to detect local differences in the reflection properties of a surface when only differences in microscale roughness occur.

In the field of computer vision and computer graphics, this issue has already been addressed and a number of models have been developed that particularly deal with the recognition of spatially-varying materials (see, e.g., Alldrin, Zickler, & Kriegman, 2008; Goldman, Curless, Hertzmann, & Seitz, 2010; Hui & Sankaranarayanan, 2017; an overview of some earlier models can be found in Lensch et al., 2005). However, several reasons speak against the idea that these kinds of models, which are generally based on an inverse optics approach, are suitable to describe the perceptual performance of a human observer. For instance, in these models the relevant information is usually obtained under strictly controlled conditions which include many restrictions and assumptions that are hardly fulfilled in everyday situations (e.g., as input to the model usually a series of images of the surface is required, taken from a fixed viewpoint under varying light directions, where the illumination is often assumed to be a distant directional light source with known position and each material is assumed to be an additive mixture of a limited set of fundamental materials). Another unrealistic aspect of such models is the kind of information that is required to estimate the reflection properties of a surface. In the aforementioned models, a material is usually represented as a BRDF (bidirectional reflectance distribution function; see Nicodemus, Richmond, Hsia, Ginsberg, & Limperis, 1977), which means that for an appropriate estimate a sufficiently large number of intensity measurements under different directions of incident and/or reflected light are required for each pixel on the surface (see, e.g., Hui & Sankaranarayanan, 2017).

**Figure 2.**
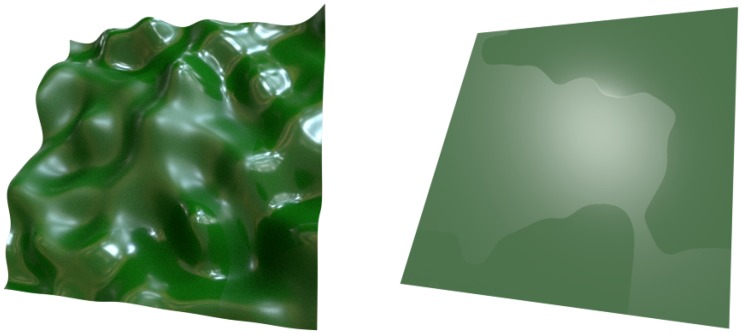
Both images show a surface of uniform greenish diffuse color that is split into two areas with different microscale smoothness. In the left image, the surface has a bumpy shape and was rendered under a real-world environment map (“Eucalyptus grove”; see Debevec, 1998). The surface is covered with a complex highlight pattern and the two areas of different smoothness differ with respect to several highlight features: Highlights in the high gloss area generally have a smaller size, sharper contours, and a higher luminance contrast than those in the low gloss area. In the right image, the surface is completely flat and a single point light source was used as illumination. Under these conditions, no distinct highlight pattern appears on the surface. However, as an alternative cue for the presence of spatially-varying materials, the prominent luminance contrast between the two adjacent smoothness areas may be used (see Figure 3).

In general, the human visual system does not seem to rely on such a point-based material evaluation but on visual cues that are extracted from larger areas of the surface (see, however, Mausfeld, Wendt, & Golz, 2014): The spatial properties of highlights (or more generally of mirror images of the illumination), such as their size, or the relative proportion of the surface that is covered with such features, as well as the sharpness of their contours, have been shown to be relevant cues for the glossiness of a surface (Beck & Prazdny, 1981; Di Cicco, Wijntjes, & Pont, 2019; Forbus, 1977; Kim, Marlow, & Anderson, 2012; Kim, Tan, & Chowdhury, 2016; Marlow & Anderson, 2013; Marlow, Kim, & Anderson, 2012; Qi, Chantler, Siebert, & Dong, 2014, 2015). In addition, the luminance contrast between the highlights and the diffusely reflecting areas, for which a comparison between different locations on the surface is needed, has been found to play a role in the perception of glossiness (Hansmann-Roth, Pont, & Mamassian, 2017; Hunter, 1975; Leloup, Pointer, Dutré, & Hanselaer, 2010; Pellacini, Ferwerda, & Greenberg, 2000). Hence, in order to detect local differences in the glossiness of a surface, such highlight features must be perceived as different across different areas on the surface (left image in Figure 2), an ability that might also be influenced by an effect that Hansmann-Roth and Mamassian (2017) describe as simultaneous gloss contrast. These authors have recently shown that the perceived glossiness at a fixed location on a surface is affected by the glossiness of neighboring areas.

**Figure 3.**
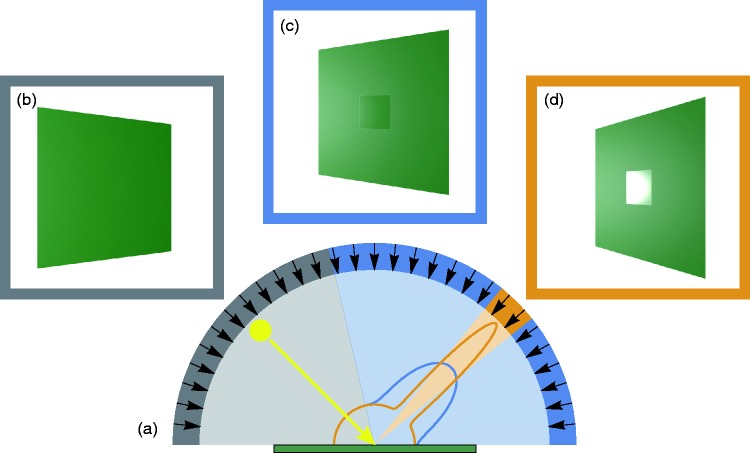
A flat surface (green) showing a spatially inhomogeneous specular reflection under a fixed orientation and a fixed direction of the incoming light (yellow arrow in (a)). The area that is occupied by the center patch has a higher microscale smoothness than the rest of the surface. The different reflective behaviors of these two areas are schematically depicted in (a) by the different forms of the cross section of the two BRDFs (see Nicodemus et al., 1977): For all possible viewing directions (black arrows), the relative amount of light that is reflected from the center of the surface to the observer is shown as a colored curve (orange for the area with higher smoothness, blue for the one with lower smoothness), where the intensity of the reflected light is represented by the distance between the point at which the incident light hits the surface (tip of the yellow arrow) and the respective point on the curve. At viewing directions where the eye exclusively receives diffusely reflected light (hemispherical parts of the two curves), the surface looks homogeneously colored (see (b) and the gray segment in (a)). When the surface is viewed from angles near the mirror direction of the incident light (orange segment in (a)), those parts with higher smoothness look considerably brighter than the remaining parts of the surface (d). From all other viewing directions (blue segments in (a)), the surface area with higher smoothness appears darker than the surrounding (c), since at these angles the specular lobe that is associated with the low gloss area (blue curve in (a)) provides higher intensity values compared to the specular lobe of the high gloss area (for a demonstration of this effect with a real glossy object, see Lensch et al., 2005, p. 62). Because realistic BRDFs have to obey the law of energy conservation (i.e., the volume enclosed by the two BRDFs in (a) must be identical), the specular lobes of different BRDFs will always show a partial overlap, at least when they share the same diffuse component. This means that the luminance contrast effect described here always occurs between areas on the same surface that only differ in specular reflection.

A further potential source of information that does not rely on the presence of distinct highlights or mirror images is the luminance contrast at the border between two areas with different microscale smoothness (right image in Figure 2) which can make the two areas appear to have different albedos (see also Hansmann-Roth & Mamassian, 2017; Sawayama, Adelson, & Nishida, 2017; Toscani, Valsecchi, & Gegenfurtner, 2017). As Figure 3 illustrates, this luminance contrast is always 0 when exclusively diffusely reflected light reaches the eye of the observer. If the eye also receives specularly reflected light, the two adjacent areas will generally differ in luminance whereby the contrast polarity is not fixed but changes in dependence on the two roughness values and the angle between the viewing direction and the dominant direction of reflection (see also Ged et al., 2010).

### Aim of the Study

The aim of this study is to examine the ability of human observers to detect differences in the glossiness of two surface areas with different microscale smoothness (Figure 2). In addition, we test how the detection performance depends on several context factors, such as the shape of the surface, the kind of illumination (point light source versus real-world environment map), the availability of motion information, the sharpness of the edge between the two areas (sharp versus blurry), and the relative spatial proportions of the two smoothness areas. Some of these context factors have already been found to have an influence on gloss perception: For instance, it was shown that the gloss impression strongly depends on the local curvature of an object (Nishida & Shinya, 1998; Olkkonen & Brainard, 2011; Vangorp, Laurijssen, & Dutré, 2007; Wendt, Faul, Ekroll, & Mausfeld, 2010) as well as on the lighting conditions (Adams, Kucukoglu, Landy, & Mantiuk, 2018; Fleming, Dror, & Adelson, 2003; Motoyoshi & Matoba, 2012; Olkkonen & Brainard, 2010, 2011; Pont & te Pas, 2006; Todd & Norman, 2018; Wendt & Faul, 2017) and the presence of motion information (Doerschner, Fleming, Yilmaz, Schrater, Hartung, & Kersten, 2011; Hartung & Kersten, 2002; Sakano & Ando, 2010; Wendt & Faul, 2018; Wendt et al., 2010). For the present task, it is to be expected that these context factors will differently affect the availability of the cues and thus also detection performance. For example, in order to make use of highlight properties for a comparison between different areas, the surface must provide a sufficiently complex highlight pattern which in general only occurs on surfaces with a complex three-dimensional (3D) geometry or under a complex illumination (Figure 2). On the other hand, the flatter the surface the more pronounced the border contrast between the two areas of different smoothness may be perceived (right image in Figure 2), especially when these areas are separated by a sharp rather than by a blurred edge. As these few examples already suggest, interactions between individual factors are also likely to occur.

## Experiment

As already mentioned, the aim of the experiment was to determine how the detection thresholds for spatially-varying surface reflection properties depends on a number of context factors. In the experiment, a single computer-generated test object was presented to the subject during each trial, and the subject was asked to indicate whether the surface appeared to be made entirely of the same material or whether it consists of areas with different reflection characteristics. We used the Unity game engine (version 2018.1.1) for the display of the stimuli and the control of the experiment.

## Methods

### Surface

**Figure 4.**
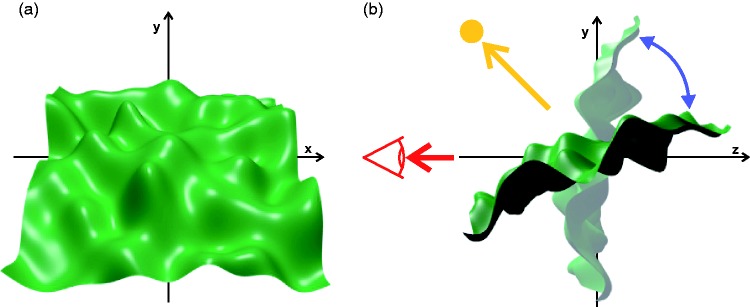
(a) The original shape of the surface (view from front). Note that in the experiment, where we tested the influence of the bumpiness on detection performance, the surface was scaled along its height dimension, such that in the extreme case a completely flat surface resulted. (b) Schematic representation of the stimulus scene (view from positive x-direction): The surface rotated back and forth between the two depicted orientations about its horizontal middle axis with a constant speed of 48°/second (blue arc). The directions to the observer and the point light are represented by a red and a yellow arrow, respectively.

The test object was a single computer-generated bumpy surface with a square base whose height profile was generated by summing a number of sine gratings according to the following equation (left image in Figure 4; see also Wendt et al., 2010):

(1) $$\begin{array}{c} y=\overset{40f-1}{\underset{k=0}{\sum }}\exp\left[-k^{2}/{\left(40f\right)}^{2}\right]\text{sin}\left[\left(x\text{cos}\left(o_{k}\right)+z\text{sin}\left(o_{k}\right)\right)\frac{\pi k}{1000}+p_{k}\right] \end{array}$$

The square base consisted of a 100 × 100 grid of equally spaced points in the (*x, z*) plane, where for both dimensions integer values between 1 and 100 were used. The *y*-coordinate represents the respective height of these points. Each of the sine gratings had a unique frequency between 0 and 2 times the maximum frequency *f* (cycles per side-length). Its orientation *o_k_* and phase *p_k_* were drawn randomly from the intervals [0, π] and [0, 2π], respectively. We used an exponential weighting function for the amplitude of the sine gratings such that higher frequencies contributed less to the overall height profile of the surface. We used a value of 3.0 for the maximum frequency parameter *f*. The resulting mesh, which consisted of 20,000 triangles, was first scaled along the height dimension *y* such that the distance between the lowest and the highest *y* value was equal to 40% of the side-length of the square base. After the mesh was imported into the Unity game engine, it was equally scaled along all dimensions such that the base had a side length of 0.2 units. For the camera settings and viewing conditions used in the experiment, this corresponds to a side-length of 5.16° of visual angle when the surface is oriented frontoparallel to the line of sight (i.e., when the global surface normal points in the direction of the observer). Note that during the experiment the height was systematically varied.

In some conditions, the surface was presented dynamically by rotating the surface back and forth in a range of 60° between two fixed orientations (at 5° and 65° relative to the *y*-axis) around its horizontal middle axis (*x*-axis in Figure 4) with a constant speed of 48°/second (see Figure 4(b)). Note that in the experiment a short adaptation period of 1-second duration during which no stimulus was visible was inserted between the presentation of two subsequent stimuli (see section “Procedure”). However, internally the rotation cycle continued during this period such that the initial orientation of the surface was not constant in each stimulus but depended on the surface’s orientation in the previous stimulus at the time a decision was submitted (i.e., the orientation of the surface in the next stimulus was shifted forward by 48° within the rotation cycle relative to the last visible orientation of the surface in the previous stimulus). In another condition, the surface was presented statically, where the global surface normal was oriented along the half-angle between the viewing direction and the direction of the point light (22.5°).

### Material of the Surface

For the material of the surface, we used the physically based standard shader from Unity with the specular setup under the opaque rendering mode. The diffuse component, or albedo, was set to a greenish color (with rgb = 0, 0.808, 0.141). The smoothness parameter was set to 1.0. However, since we used two-dimensional glossmaps under all stimulus conditions to realize spatially-varying smoothness areas, this parameter only served as a factor for the values stored in these glossmaps.

**Figure 5.**
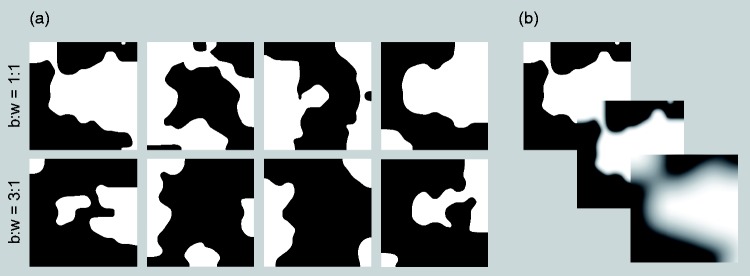
(a) The eight basic textures used for the glossmaps, both for a black to white pixel proportion of 1:1 (top row) and 3:1 (bottom row). (b) The same basic glossmap shown with different degrees of edge sharpness. In the upper left image, the original glossmap with sharp edges is shown. The corresponding glossmaps with blurred edges were generated by applying Gaussian smoothing with a standard deviation of 10 (middle image) or 50 (bottom image).

As basic glossmaps, we generated eight different textures consisting of irregular black and white structures that look similar to the black and white patched coat of Holstein Friesian cattle (see Figure 5(a)). These textures were generated with a procedure that was mainly based on Perlin noise (Perlin, 1985; see Appendix A for a detailed description of the construction process). Four of them were created such that the black and white areas had the same number of pixels (top row in Figure 5(a)) while the other four textures had a black to white proportion of 3:1 (bottom row in Figure 5(a)). Smoothness values for the glossmaps between 0.2 and 0.8 were used. These actual smoothness parameters replaced the black and white patches, respectively, of the basic maps. To approximate a perceptually equidistant scale, we used a modified version of the smoothness scale implemented in Unity, where *scaled smoothness = original smoothness^1/1.77^* (for more details, see Wendt & Faul, 2017). If not otherwise stated, the term “smoothness” refers to this scaled smoothness parameter.

In the experiment, we also investigated how the detection threshold is influenced by the sharpness of the edge between the two different smoothness areas. Sharp edges often occur on surfaces made of different materials (see the left image in Figure 1), while blurred edges are often caused by impurities or external influences on surfaces of uniform material (see the middle and right images in Figure 1). Glossmap textures with blurred edges were generated by applying Gaussian smoothing to the original images, using the MATLAB function *imgaussfilt* with standard deviations of 10 and 50, respectively (see Figure 5(b)). Planar texture mapping was used to project the final glossmap to the surface. The backside of the surface had a diffuse black color (rgb = 0, 0, 0).

### Lighting

We used two different kinds of lighting, namely, a single point light and a real-world illumination map that includes interreflections from the environment. In the point light condition, the light source was located at position (0.0, 3.5355, –3.5355), that is, to the top front of the surface with a distance of 5 units to the center of the object (Figure 5(b)) and an angle of 45° relative to the viewing direction (negative *z*-axis in Figure 4(b)). The color of the light was white (rgb = 1.0, 1.0, 1.0) with an intensity of 2.0. The effective range, that is, the distance beyond which the light energy drops to 0, was set to 10.0 units. For the remaining light parameters, the default settings were used, which includes the use of soft shadows.

For the second lighting condition, we used the “St. Peter’s” environment map from Debevec’s Light Probe Gallery (Debevec, 1998), which provides a full spherical panorama of the inside of the St. Peter’s Basilica in Rome. The HDR (high dynamic range) image (from www.pauldebevec.com/Probes/) was imported to Unity as texture type “Default” with the texture shape “Cube.” The mapping was set to “Mirrored Ball (Spheremap)” with the convolution type “Specular (Glossy Reflection).” The wrap mode was set to “Clamped” and the filter mode to “Trilinear.” The maximum size was set to 1,024 pixels and the compression to “High Quality.” The remaining settings were unchanged. In the “Environment Reflections” section of Unity’s lighting window, the map was inserted with the compression option “Uncompressed” and an intensity multiplier of 0.414. This latter parameter value was chosen such that both the point light source and the environment map produced reflections on a reference surface with approximately equal mean luminances, which was measured in both cases using a fixed orientation of the reference surface (which was tilted 22.5° backwards relative to a starting orientation where the global surface normal points to the direction of the observer) with a spatially uniform smoothness of 0.3. The height profile of the surface was scaled with the factor 0.5 in these cases. For both kinds of illumination, an additional ambient component was used with achromatic color rgb = (0.6, 0.6, 0.6).

### Apparatus and Viewing Conditions

A color calibrated TFT monitor (EIZO CG243W) was used in our experiments to display the stimuli. The screen had a width of 52 cm and a height of 32.5 cm with a resolution of 1,920 × 1,200 pixels. The CIE 1931 color coordinates of the maximum white with rgb = (1.0, 1.0, 1.0) were xyY = (0.324, 0.324, 115.8). The stimuli were always presented stereoscopically by means of a mirror stereoscope (SceenScope) to enhance the perception of gloss (Wendt, Faul, & Mausfeld, 2008). The total distance between the screen and the eyes of the observer was 50 cm. The two monocular half-images of the stimulus were computed with two perspective cameras located in the scene at positions (–0.03, 0, –1) and (0.03, 0, –1) for the left and the right eye, respectively. The original settings for both camera components were 60° for the field of view and 0.5 and 3.0 units for the near and the far clipping plane, respectively. However, since we used the off-axis projection according to Kooima (2008), these values were generally changed by a script as soon as the experiment started. As further camera settings we chose a black background color (rgb = 0, 0, 0) and enabled HDR rendering. We used tonemapping to rescale the HDR values such that the images could be displayed on our LDR display device. To this end, we activated the “Color Grading” effect of Unity’s post-processing stack where we used the “Neutral” tonemapper with the default settings (“Black In” = 0.02, “White In” = 10, “Black Out” = 0, “White Out” = 10, “White Level” = 5.3, and “White Clip” = 10). The two monocular half-images had a size of 25% of the width and 50% of the height of the screen and were presented side by side in the center of the screen.

### Procedure

In each trial, a single test object was presented to the subjects where the surface consisted of two areas that generally differed in microscale smoothness (see Figure 2). In a two-alternative forced choice (2AFC) task, the subjects had to indicate whether or not the stimulus appeared to have a spatially homogeneous material. A double random staircase procedure (Cornsweet, 1962; Kingdom & Prins, 2010; Meese, 1995) was used to determine the detection threshold for a spatially heterogeneous material. The detection threshold is defined as the point of subjective equality (PSE) between a surface with spatially homogeneous and a surface with spatially heterogeneous material.

To this end, one of the two areas was held constant at a baseline smoothness level *b* while the smoothness level of the second area varied between *b* (where the entire surface has the same smoothness level) and *b* + 0.4 in steps of 0.01. The two staircases S_A_ and S_B_ started at opposite ends of this smoothness interval, that is, one at baseline level *b* and the other one at smoothness level *b* + 0.4, with a step size of 0.2. Whenever a reversal occurred in a staircase, that is, whenever the judgment changed from “homogeneous” to “heterogeneous” or vice versa, the step size was halved and the resulting smoothness value was then rounded to the second decimal place (since the glossmaps were pre-rendered with a step size of 0.01 for the variable smoothness area). For each step in a trial one of the two staircases, S_A_ or S_B_ was randomly chosen as the currently active one. The trial ended when either both staircases had at least six reversals or when the total number of steps exceeded 50. In either case, the mean of the smoothness values of the variable area observed in the last six steps was taken as the threshold value.

**Figure 6.**
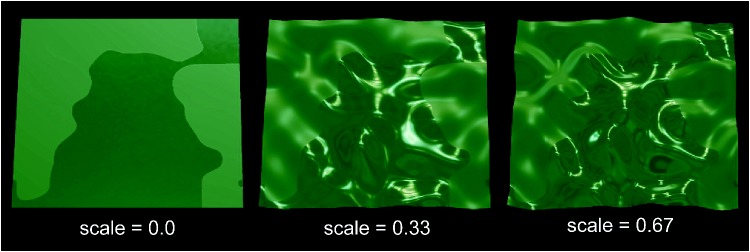
Screenshots of three example stimuli (each one showing the right monocular half-image of the stereo pair). We examined three different shapes for the surface which only differed in the scaling of the height profile. All stimuli are shown under otherwise identical conditions, namely, under static presentation and the real-world environment map “St. Peter’s,” with a spatial proportion of 1:1 between the two smoothness areas which are separated by a sharp edge. The two different reflection areas have smoothness values 0.4 for the baseline *b* and 0.8 for the remaining parts of the surface, respectively.

In each trial, the detection performance was tested under a different combination of context factors. The context factors were surface shape (between flat and bumpy in three levels with scaling factors 0, 0.33, and 0.67, respectively, see Figure 6), the light field (single point light vs. a real-world environment map), the availability of motion information (rotating vs static presentation of the surface), the sharpness of the edge between the two areas of different smoothness (three levels from sharp to blurry, see Figure 5(b)), the relative spatial proportions of the areas (with the two levels 1:1 and 3:1, see Figure 5), and the baseline level *b* of the microscale smoothness (with the two levels *b* = 0.2 and 0.4). The entire set of 144 different condition combinations was tested 3 times, resulting into a total of 432 stimuli that were presented in random order. As part of the instruction, a small set of four different example stimuli had to be completed by the subject prior to the experiment while the investigator was present.

Using a single glossmap bears the danger that the subjects quickly learn the exact locations of areas of different smoothness and focus only on these parts of the surface. To encourage the subjects to base their material judgment on the entire surface, one of 16 different glossmap structures (four glossmap textures of a kind, see Figure 5(a), each in four different orientations between 0° and 270° in steps of 90°) was randomly selected in each step.

The keys to be used were indicated by the text “homogeneous or heterogeneous?” together with a left and a right arrow symbol, respectively, underneath the stimulus. During a short adaptation period of 1 second after each trial, only the response text field and a trial counter at the top of the stimulus were visible on an otherwise black screen.

As our goal was to explore the general detection performance under approximately realistic viewing conditions, no time restrictions were imposed on the subjects to complete a trial. However, the subjects were informed that the time and the number of steps they needed to complete a trial were recorded. The subjects could pause and continue the experiment by pressing the space bar. During the pause, the stimulus disappeared and “PAUSE” was shown at the center of the screen. After the pause, the current trial was stopped and restarted from the beginning. On average, the subjects needed about 11 hours to complete the experiment, distributed over 5 to 6 sessions.

### Subjects

Six subjects participated in the experiment, including one of the authors (G. W.), who had normal or corrected to normal visual acuity. This study was conducted in accordance with the Code of Ethics of the World Medical Association (Declaration of Helsinki) and informed consent was obtained for the experimentation with human subjects.

## Results

As a measure for the detection threshold, we used the difference in smoothness (Δsmoothness) between the two areas of the surface at the PSE. The overall average threshold was 0.083, the average threshold values for the single condition combinations ranged from 0.0064 (for the combination “point light” × “spatial smoothness proportion of 1:1” × “sharp edge” × “base smoothness level 0.4” × “static surface” × “flat surface shape”) to 0.1806 (for the combination “environment lighting” × “spatial smoothness proportion of 3:1” × “strongly blurred edge” × “base smoothness level 0.2” × “dynamic surface” × “bumpy shape”).

A six-way analysis of variance (ANOVA) was performed on the data, using the threshold values as dependent variable and all six factors “lighting,” “spatial proportion,” “edge blurring,” “base smoothness,” “motion,” and “shape” as independent variables. Table B1 (see Appendix B) shows the results for all main effects, all first-order interaction effects, and all significant interaction effects of higher order. In the same way, we evaluated the decision time data, that is, the average time per step within each trial that was needed by the subjects to submit a response (see Table C1 in Appendix C).

### Threshold Results

**Figure 7.**
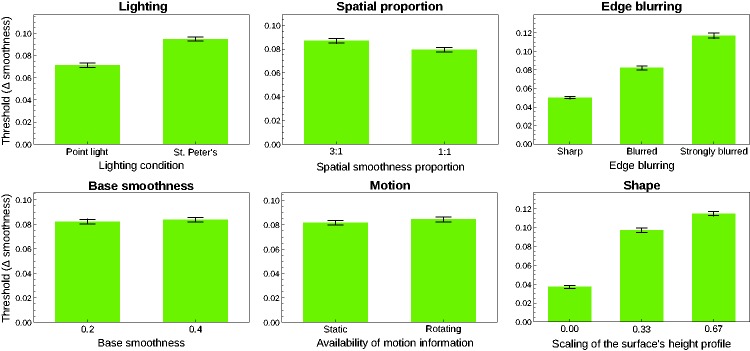
Main effects of the six factors with respect to the threshold results. The data are averages across all six subjects. Error bars represent *SEM* in both directions.

With respect to the threshold data, all but the factors “motion” and “base smoothness” had a significant effect (see Figure 7). Specifically, the detection performance was significantly better with a point light source than with the real-world illumination “St. Peter’s” (top left diagram in Figure 7). Thresholds were also lower when the spatial proportions of the two smoothness areas on the surface were balanced than when the high smoothness area occupied only 25% of the surface. The degree of edge blurring (top right diagram in Figure 7) and the bumpiness of the surface (bottom right diagram in Figure 7) also had a strong influence on the detection threshold: The sharper the edge between the two smoothness areas and the less bumpy the shape of the surface, the better the detection performance.

**Figure 8.**
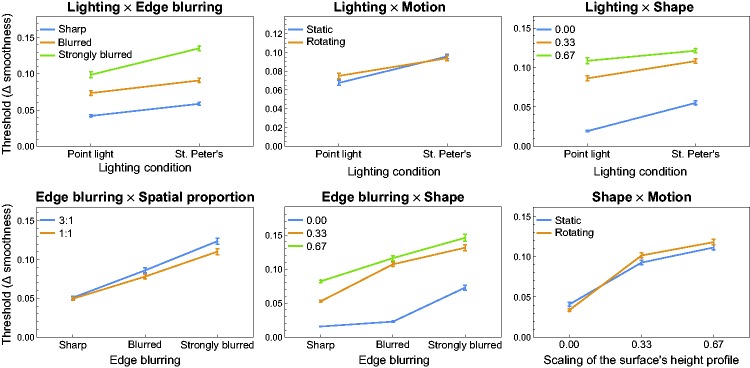
Six significant first-order interaction effects with respect to the threshold results. The data are averages across all six subjects. Error bars represent *SEM* in both directions.

**Figure 9.**
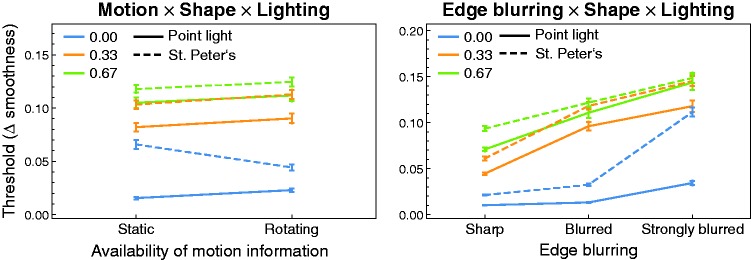
Two significant second-order interaction effects with respect to the threshold results. The data are averages across all six subjects. Error bars represent *SEM* in both directions.

Six of the first-order interaction effects between the factors were significant (see Figure 8). The detection threshold was disproportionally higher for strongly blurred edges when a real-world illumination map was used (top left diagram in Figure 8), or when the spatial proportion of the two smoothness areas was unbalanced (3:1, see bottom left diagram in Figure 8). Detection performance was more improved for a flat surface compared to the two other shape levels, when a point light was used instead of an illumination map (top right diagram in Figure 8). When the smoothness areas were separated by a strongly blurred edge, the detection performance decreased more strongly with a flat surface than for surfaces with a more or less bumpy shape (bottom middle diagram in Figure 8). We further found significantly lower thresholds for a flat surface when in addition the surface was presented dynamically instead of statically, while for bumpy surfaces this trend was reversed (bottom right diagram in Figure 8). Although the main effect for the factor “motion” was not significant, there was a significantly better detection performance for a static surface compared to a rotating surface when a point light was used as the light source (top middle diagram in Figure 8). Noticeable second-order interaction effects are shown in Figure 9 and will be discussed in detail in the Discussion section.

### Decision Time Results

On average, the subjects viewed each stimulus for 1.76 sec before submitting their decision. For the 144 different condition combinations the average decision time ranged from 0.935 sec (for the combination “point light” × “spatial smoothness proportion of 1:1” × “blurred edge” × “base smoothness of 0.4” × “static surface” × “flat surface shape”) to 4.236 sec (for the combination “environment lighting” × “spatial smoothness proportion of 1:1” × “strongly blurred edge” × “base smoothness level 0.4” × “rotating surface” × “strongly bumpy surface shape”).

Note that the average decision times should be treated with some caution: It is reasonable to assume that the time the subjects needed to decide whether or not there is a material difference depended on the actual smoothness difference between the two surface areas. This decision will generally take longer near and below the threshold than for stimuli that are well above the detection threshold. Hence, especially for those initial steps of a trial that started at a maximum smoothness difference between the two surface areas, particularly low decision times were to be expected with the consequence that the entire set of decision times of a condition combination may not be normally distributed but negatively skewed. In addition, since for the average decision time all steps of a trial were taken into account, these temporal “outliers” may have lowered the average value—a bias that would be the stronger the smaller the number of steps required for a trial. In order to check whether there were systematic differences in the number of steps between different context conditions, we analyzed the respective data and found that on average the subjects needed 27.73 steps per trial, with mean values for the 144 different condition combinations ranging from 24.72 to 33.5 steps. Although we found significant main effects of the two context factors “edge blurring” (F(2,2448) = 40.57, p < .001, *η_p_*^2^ *=* 0.0321) and “base smoothness” (F(1,2448) = 11.03, p < .001, *η_p_*^2^ *=* 0.0045) on the step numbers, the effect sizes were rather small and the absolute average values between the single levels of each factor differed only slightly: For the three different levels of the factor “edge blurring” these average step numbers ranged from 26.74 to 29.22 (the more blurry the edge the more steps were required) and for the two different levels of the factor “base smoothness” the average step numbers were 27.34 (for *b* = 0.2) and 28.13 (for *b* = 0.4), respectively. Furthermore, three higher order interaction effects turned out to be significant, which were also characterized by rather small effect sizes (with *η_p_*^2^ *<* 0.0048). This suggests that the above-mentioned bias was quite similar between the different conditions and did not seem to affect the general trends we have found in the decision time data.

**Figure 10.**
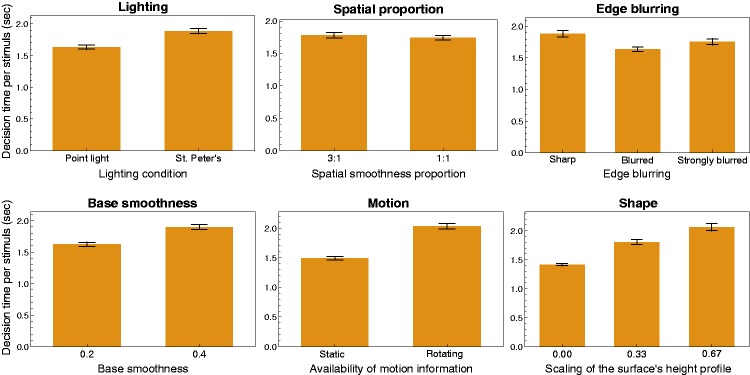
Main effects of the six factors on the decision time, averaged across all six subjects. Error bars represent *SEM* in both directions.

The main effects on the decision time found with a six-way ANOVA are depicted in Figure 10 (see also Table C1): With the exception of the factor “spatial smoothness proportion” (top middle diagram in Figure 10), all factors had a significant main effect. For the two factors “lighting” and “shape,” the decision time seems to be correlated with the threshold data in the way that low thresholds are accompanied by small decision times and vice versa. A notable exception could be found for the factor “edge blurring”: Although sharp edges between areas of different smoothness generally led to a considerable decrease in the thresholds, the subjects needed on average significantly more time to judge stimuli with sharp edges than those with blurred edges. The remaining two factors “base smoothness” and “motion,” that is, those factors that did not had any significant influence on the thresholds, had significant main effects on decision time: Statically presented surfaces were judged considerably faster than rotating surfaces and surfaces with a base smoothness of 0.2 faster than those with a higher base smoothness level of 0.4.

**Figure 11.**
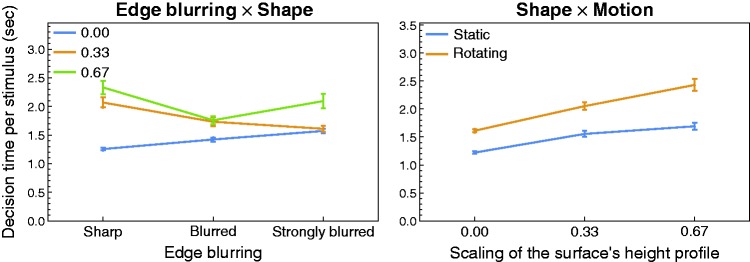
Graphical representation of two significant first-order interaction effects with respect to the decision time results. The data were averaged across all six subjects. Error bars represent *SEM* in both directions.

Two of the first-order interaction effects were also significant (see also Table C1), one being the interaction between the factors “edge blurring” and “shape”: While for a completely flat surface the decision time seems to systematically increase with the degree of edge blurring, this trend is rather reversed for the two bumpy shape conditions (left diagram in Figure 11). The other significant interaction effect was the interaction between the factor “shape” and the availability of motion information. Under both levels of the motion factor, the decision time systematically increases with increasing bumpiness of the surface, however, for the rotating stimuli this increase was steeper than with statically presented surfaces (right diagram in Figure 11).

## Discussion

In this study, we tested the ability of human observers to detect spatial differences in the glossiness of a surface in dependence on six context factors. In the following, we will discuss the influence on the detection performance separately for each of these factors.

### Motion

It may be surprising that, on average, there was no statistically significant difference in the detection performance for statically presented and rotating surfaces. A priori, we expected the rotating surface to provide more task relevant information, especially when the real-world illumination is used at the same time, as we assumed that due to object rotation larger areas of the environment would be reflected to the observer and that this would increase the chance to capture a section of the environment that produces an especially diagnostic pattern on the surface. As can be seen in the corresponding interaction diagram (top middle diagram in Figure 8), this was generally not the case, as there is no significant difference in threshold with a rotating and a statically presented surface under the St. Peter’s illumination map. However, if the factor “shape” is additionally taken into account, one can see in the corresponding diagram (left diagram in Figure 9) that our initial assumption actually holds for a completely flat surface, that is, for that shape which reflects the smallest (but therefore undistorted, see Fleming, Torralba, & Adelson, 2004) section of the environment to the observer (see the dashed blue line in the left diagram of Figure 9). For the two remaining levels of the shape factor, that is, for surfaces that show some local curvatures (with scaling factors 0.33 and 0.67, respectively) a slight trend in the opposite direction can be seen, that is, the detection performance was always slightly better for static surfaces, irrespective of the kind of illumination. At least for the point light, this latter finding is not surprising, because the respective static stimuli were constructed in a way that the surface had an ideal orientation relative to the light direction and the viewing direction (see Figure 4(b)). It was therefore almost guaranteed that diagnostic information was available on the surface.

Regarding the decision time data, subjects needed on average significantly more time for their decision when the surface was presented with a rotation compared to a static surface (see the bottom middle diagram in Figure 10). On average, the rotating stimuli were viewed for about 2.03 seconds (compared to 1.49 seconds in the static case) which means that the full sequence of different orientations of the surface (within the 60° cycle, see Figure 4(b)) was viewed more than 1.6 times. This suggests that the subjects took the opportunity to wait for an orientation of the surface that provides the most diagnostic features for the presence of spatially-varying materials. As we have just seen, this strategy seemed to be successful at least in cases with a flat surface under a complex illumination map (see the bottom right diagram in Figure 8). However, such an advantage of longer viewing times for rotating stimuli was not found when the surfaces had a bumpy shape: Although in these cases, where at certain orientations of the surface relevant information about its spatial material distribution may have been blocked from view, even more time was needed for a decision (up to 2.43 seconds, see the right diagram in Figure 11), this did not improve the detection performance (bottom right diagram in Figure 8).

### Lighting

The fact that the scenes were constructed in a way that created almost ideal conditions for the point light source has probably contributed to the result that the detection performance was on average considerably better and the decision time significantly shorter (see top left diagram in Figure 10) with a point light than with a complex illumination map. It is to be expected that the result with a complex illumination depends also on the specific environment map used. Relevant properties of the map could be the presence, the extension, and spatial distribution of direct light sources (or generally of bright spots) in the map (see also Wendt & Faul, 2017).

**Figure 12.**
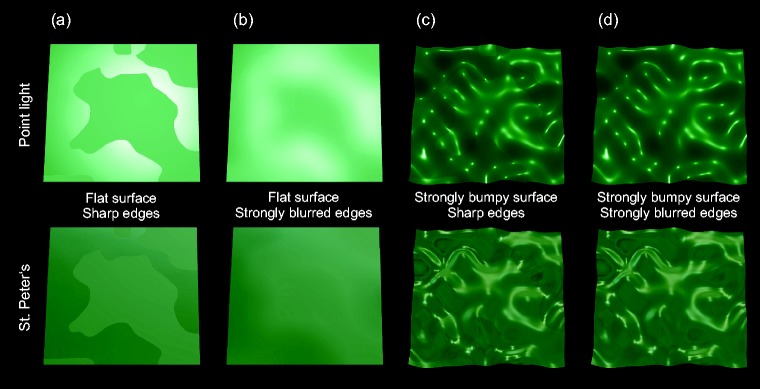
Some example stimuli from the experiment used to illustrate the effects of the interaction between the factors “shape,” “edge blurring,” and “lighting” on the detection performance (shown are only the right half-images of the respective stereo-pairs). Columns (a) and (b) show a completely flat surface, columns (c) and (d) a strongly bumpy surface. The two different smoothness areas of the surfaces in columns (a) and (c) are separated by a sharp edge while in columns (b) and (d) this edge is strongly blurred. The stimuli in the top row were rendered using a point light. For the stimuli in the bottom row the complex environment map “St. Peter's” (see Debevec, 1998) was used as illumination. The remaining factors were kept constant: All stimuli were taken from the static presentation condition and the two smoothness areas have a balanced spatial proportion (1:1). The base smoothness level was 0.4 while the high smoothness area was set to a value of 0.7.

However, the difference in detection performance between the two lighting conditions also varies with other context factors and there were some cases where this difference was comparatively small. For instance, as can be seen in the right diagram of Figure 9, the combination of a strongly bumpy surface with strongly blurred edges between the smoothness areas leads to a nonsignificant threshold difference of less than 0.0049 smoothness units between the two lighting conditions (see also Figure 12(d)). Under these context conditions, observers have to base their judgment mainly on the difference in perceived highlight features such as their sharpness, their size, and their intensity, and in this case these features are similar under both kinds of illumination.

With a flat surface, however, there are no distinct highlight patterns on the surface and the only information that can be used to detect differences in the surface material is the luminance contrast between areas of different smoothness (see also Figure 3). Although this luminance contrast is considerably stronger under the point light condition, the detection thresholds are rather similar in the two lighting conditions, if there is a sharp edge between the smoothness areas (with a difference of 0.011 smoothness units, see the right diagram in Figure 9; also compare the two stimuli in Figure 12(a)). Indeed, the detection of spatial material differences seems to be reduced to edge detection in these cases, a mechanism that on an absolute scale leads to rather low detection thresholds, even for the comparatively small luminance contrasts produced by the real-world illumination map.

However, the detection performance dramatically decreases under the real-world illumination compared to the point light condition when areas of different smoothness are separated by a strongly blurred edge (with a difference of 0.077 smoothness units, see the right diagram in Figure 9 and Figure 12(b)). This is in line with the finding that the sensitivity to detect luminance contrasts is reduced with blurred edges (Hood, 1973). Hence, considerably larger differences in smoothness are required (see Figure 3) to compensate for the comparatively low intensity in the relevant sections of the “St. Peter’s” illumination map.

While the static flat surface under the point light condition always led to a vivid impression of a glossy surface, this was not the case for the same surface under the “St. Peter’s” illumination map. This indicates that the detectability of highlight disparity information from the luminance distributions of the stereoscopically presented surfaces is reduced with the illumination map (Wendt et al., 2008). The subtle luminance variations that result in this condition may be interpreted as a surface texture rather than a mirror image of the environment. The strong reduction of the thresholds with dynamic presentation (see the dashed blue line in the left diagram of Figure 9) suggests that this ambiguity might be resolved by motion induced cues (Doerschner et al., 2011; Sakano & Ando, 2010).

### Shape

Our data suggest that in general the detection performance is significantly higher and the decision time systematically shorter (see bottom left diagram in Figure 10) when the judgment can be based on the luminance contrast between adjacent areas of different smoothness and not on differences in certain highlight features (cf., e.g., Figure 12(a) with Figure 12(c) or 12(d)). The availability of these two cue classes for the detection of material differences is mainly modulated by the 3D geometry of the surface (see also Figure 6): At least under the conditions realized in the present experiment, a completely flat surface predominantly provides luminance contrast information that is caused by different smoothness values in the two areas (see Figure 3). Especially in combination with a sharp edge between the smoothness areas, rather low detection thresholds were found with such stimuli (Δsmoothness of about 0.016; see bottom middle diagram in Figure 8). Bumpy surfaces, on the other hand, usually show more or less complex highlight patterns (see also Figure 2) which seem to provide much less diagnostic information for the detection of material differences (bottom right diagram in Figure 7).

Although the luminance contrast cue is also available on bumpy shapes, it is considerably less pronounced than on flat surfaces and it is therefore much more difficult to detect the sharp edge between the areas of different smoothness on a bumpy than on the flat surface (cf. Figure 12(a) and (c)). The reduced luminance contrast is caused by at least two factors: First, as illustrated in Figure 3, the magnitude of the contrast depends on the geometrical relationship between the light direction, the viewing direction, and the orientation of the surface normal. Since the orientation of the normal varies with position on a bumpy surface, the magnitude of the luminance contrast also generally varies along the edge between the two areas of different smoothness. Second, while the luminance variations that appear on a flat surface are to a large part determined by the microscale smoothness (see Figure 12(a) and (b)), the spatial luminance distribution on a bumpy surface also depends on further effects: Due to the complex 3D geometry with a variety of local curvatures, not only the complex highlight structure but also diffuse shading and self-shadowing contribute to the intensity pattern of the surface. The presence of this complex intensity pattern may then interfere, as a kind of “noise,” with the detection of a luminance contrast edge between adjacent smoothness areas.

Hence, the considerably lower detection performance as well as the longer decision times with bumpy surfaces may not only result from the presence of a less effective cue (i.e., a complex highlight pattern) but also from impairing the detectability of a more effective cue (i.e., a luminance contrast border).

### Edge Blurring

The threshold data show a systematic decrease in the detection performance with an increase of edge blurring between the two adjacent areas of different smoothness. Some potential reasons for this result have already been discussed in previous sections. For instance, the sensitivity to detect luminance differences between the areas of different smoothness might be reduced with blurred edges (Hood, 1973). Furthermore, in the case of bumpy surfaces, the visual system may rely more on highlight properties than on luminance contrast information when the edge is blurred, and the former cue class seems to be less accurate (cf. Figure 12(c) with Figure 12(d)).

Another reason may result from the unequal distribution of smoothness values between the respective glossmaps. With a sharp edge, the glossmap contains only two different smoothness values, one at the baseline level *b* (see the top image in Figure 5(b)) and the other at *b* + Δsmoothness. With a blurred edge, however, the glossmap contains further smoothness values that lie between these two extreme values (see the middle and bottom images in Figure 5(b)). This has two consequences for the areas containing the two extreme values: The absolute number of pixels in these areas decreases and the distance between these areas increases with increasing edge blurring. Since it is plausible that the detection performance is enhanced when the sizes of the areas that comprise the maximum difference in perceived glossiness (which correspond with the two extreme values) are larger and the distance between these areas is smaller, this may have contributed to our result.

With respect to decision times, it might be surprising that they are, on average, significantly higher for stimuli with sharp edges than for those with blurred edges (see the top right diagram in Figure 10) although the detection performance with sharp edges was considerably higher. However, as one can see in the left diagram of Figure 11, this trend only holds for bumpy surfaces. In the previous section, we have argued that the detectability of a sharp edge is considerably decreased in bumpy surfaces due to the presence of luminance “noise.” It is therefore plausible that an observer needs more time to detect a luminance edge in bumpy than in flat surfaces.

### Spatial Proportion of Areas of Different Smoothness

On average the detection performance was found to be slightly better and the decision time slightly shorter when the two areas of different smoothness had a spatial proportion of 1:1 instead of an unbalanced proportion of 3:1 (see the top middle diagrams in Figures 7 and 10, respectively). Our first intuition was that this might be due to the fact that the edge between the two smoothness areas was generally longer under the balanced condition (with an average length of 1,519.25 pixels for the four different glossmap textures, see the top row in Figure 5(a)) than under the 3:1 proportion condition (with an average length of 1,223 pixels, see the bottom in Figure 5(a)): Simply because pixels that belong to a luminance edge are more frequent, a material difference should be easier to detect in stimuli with a balanced spatial proportion, especially when the two smoothness areas are separated by a sharp edge. However, as can be seen in the bottom left diagram in Figure 8, which illustrates the interaction between the factors “edge blurring” and “spatial proportion,” there is no statistically significant difference between the 1:1 and the 3:1 proportion when a sharp edge is present.

**Figure 13.**
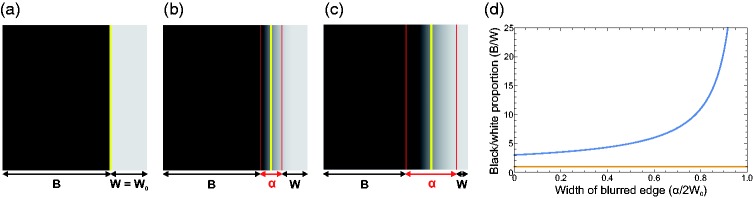
Schematic illustration of how the actual spatial proportion of black (B) to white (W) pixels depends on the width of the blurred edge (α). (a) The glossmap texture in its original form with a sharp edge where the black to white proportion is 3:1 (for demonstration purposes we use a simple bipartite texture for the glossmap). (b) and (c) With increasing blurring the edge becomes wider and the numbers of black and white pixels become smaller accordingly. For unbalanced glossmaps, the actual spatial proportion of the remaining black to white pixels deviates more and more from the original proportion with increasing width of the blurred edge (see the blue curve in (d)). For balanced glossmaps, however, the black to white proportion stays constant, irrespective of the amount of blurring (orange curve in (d)).

The fact that such a difference in detection threshold only occurs with blurred edges rather suggests an explanation in terms of the extreme values in the glossmaps (see the last section “Edge Blurring”): We found that the actual proportion of black to white pixels (that is, the two extreme values, representing the low gloss and the high gloss areas of the surface, respectively) is strongly influenced by the degree of edge blurring when the sizes of the areas in the original glossmap are unbalanced (see also Figure 13 where we present the results of a simulation using a simple bipartite field as glossmap texture). With sharp edges, the black to white proportion is as intended, that is, 1:1 and 3:1, respectively for the two levels of the factor “spatial proportion.” With blurred edges, these proportions were on average 1.001:1 for the balanced but 3.574:1 for the unbalanced condition, which already deviates noticeably from the original proportion for the unbalanced condition (note that for the analysis a black pixel was defined as belonging to the bottom 10% and a white pixel to the top 10% of the intensity range of the respective glossmap). With strongly blurred edges, this deviation in the unbalanced condition increases further with an average proportion of black to white of 10.888:1 (while the corresponding proportion in the balanced condition was on average 0.92:1). Hence, it is plausible that the weaker detection performance in stimuli with an unbalanced spatial proportion is due to the strong decrease of the size of the high gloss area (represented by the white pixels) in relation to the size of the low gloss area (represented by the black pixels) with increasing edge blurring: A comparatively small high gloss area that does not stand out sharply from its surroundings, might be hard to detect—which also explains the longer viewing times in the unbalanced condition.

### Base Smoothness

No statistically significant main effect of the factor “base smoothness” on the threshold was found. This suggests that the sensitivity of the visual system to detect spatial differences in the glossiness of a surface does not depend on the position of the base level on the smoothness scale (see the bottom left diagram in Figure 7). Since this smoothness scale was constructed as a perceptually equidistant scale where equal differences on the scale correspond to equal differences in perceived glossiness (see section “Material of the Surface”), this result seems to confirm that this scale has the desired property. However, the finding that there was a significant main effect of this factor on decision time (see the bottom left diagram in Figure 10), with an advantage for stimuli at a lower smoothness level, currently lacks a meaningful interpretation.

### General Notes

One may ask whether in this study it was necessary to use objects as stimuli whose surfaces were split into two different spatial areas with different materials and whether the same results could have been obtained by measuring just-noticeable differences (jnd) using two separate surfaces instead. Although this remains an empirical question, we assume that this may depend on the specific set of context conditions: With both methods, similar results can be expected for stimuli that comprise complex highlight patterns, as they are caused by surfaces with complex curvatures (see Figure 6). However, for surfaces where luminance contrasts play the major role in the detection of material differences, the results may differ: For instance, we found extremely low thresholds with flat surfaces, especially when the two smoothness areas were separated by a sharp rather than by a blurred edge. When two separate surfaces were used, however, it would not even be possible to apply the attribute “edge blurring” to the stimuli. In addition, luminance differences between two separate surfaces may not necessarily be interpreted as differences in the material, but as being caused by different illuminations or as shadowing effects, especially when the stimuli are presented statically. Hence, since the aim of this study was to measure thresholds for the detection of spatially-varying materials on the same surface, the best way to avoid such potential problems was to use a stimulus that simulates a single surface with exactly these heterogeneous reflection properties.

## Conclusions

We examined the ability of the visual system to detect spatial differences in the glossiness of a surface in dependence of several context factors. Our results indicate that the visual system can make use of two different cues for this task: We found that the luminance contrast between areas of different microscale smoothness provides a highly effective cue. If, under favorable context conditions, this source of information is available in an unadulterated form (e.g., on a perfectly flat surface with a sharp edge between adjacent smoothness areas), material differences are detected comparatively fast and the thresholds are extremely low. As another potential cue, the visual system can rely on certain highlight features, such as their size, intensity, and sharpness. However, our results suggest that the visual system is in general much less sensitive to differences in these highlight features between areas of different microscale smoothness than to differences in lightness. In addition, the presence of a complex highlight pattern, which is usually caused by surfaces with complex 3D geometries, seems to reduce the detectability of luminance contrasts.

## Figures and Tables

**Figure A1.**
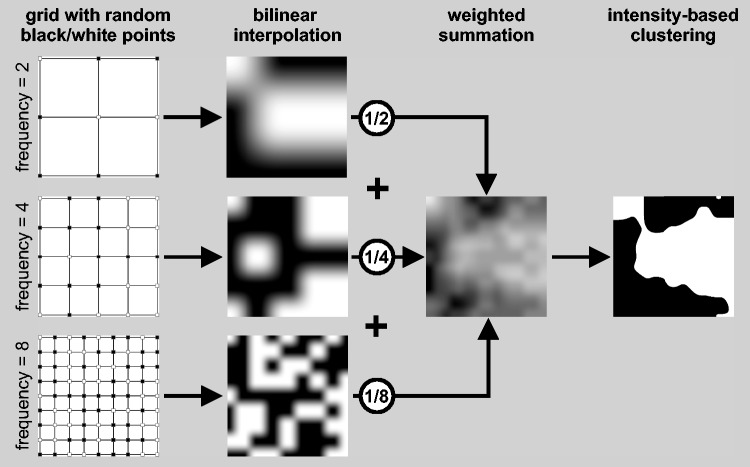
Schematic representation of the procedure used to generate the basic textures for the glossmaps (see text for details).

## Appendix A—Generation of the Glossmap Textures

The glossmaps were generated with MATLAB. The 512 × 512 pixel maps were exported as 32-bit RGBA PNG images, where the alpha channel was used to store the spatially-varying smoothness values. The RGB layers were all set to a spatially constant value of 0.5. As basic maps we generated eight different textures consisting of irregular black and white structures (see Figure 5) using a procedure that to a large part was based on Perlin noise (Perlin, 1985; see Figure A1): For each texture, we started with three two-dimensional images of 513 × 513 pixels with equally spaced points arranged in a grid (the addition of an extra row and column to the image was needed in the generation process to have a full grid). The three images differed in the number of the square grid cells, which were 4, 16, and 64 (corresponding to a frequency of 2, 4, and 8, respectively, see the left column in Figure A1). An intensity value of either 0 or 1 was randomly assigned to each of the grid points. In the next step, the pixels within each grid cell were also assigned an intensity value, which resulted from a bilinear interpolation of the intensities of the four corner points of the cell, using a smoothing function as described in Perlin (2002; see second column in Figure A1). The three frequency images were then additively combined into one image with weights that were the multiplicative inverses of their frequencies (third column in Figure A1; the excess 513th row and column of each image were ignored at this point, so that a texture with a size of 512 × 512 pixels remained). In the final step, the resulting cloudy grayscale image was transformed into a black and white image with pre-defined relative pixel proportions for the two colors (in our experiment, we used either a black to white proportion of 1:1 or of 3:1, see Figure 5). To this end, we examined the intensity histogram of the grayscale image and determined the intensity threshold at which the total pixel numbers to the left and right of this threshold met the pre-defined proportion. Pixels with intensities less than or equal to this threshold were set to black, the remaining pixels were set to white (right column in Figure A1).

## Appendix B—ANOVA Results for the Threshold Data

**Table B1.** Results of the Six-Way Analysis of Variance of the Threshold Data, Using All Six Experimental Factors: Lighting Condition (“Lighting”), Spatial Smoothness Proportion (“Proportion”), Edge Blurring (“Blurring”), Base Smoothness (“Smoothness”), Type of Surface Motion (“Motion”), and Bumpiness of the Surface’s Shape (“Shape”).

Source	Sum of squares	*df*	Mean squares	*F*	*p*	*η_p_* ^2^
Lighting	0.358	1	0.358	150.62	<.001	0.0579
Proportion	0.037	1	0.037	15.70	<.001	0.0064
Blurring	1.920	2	0.960	403.54	<.001	0.2479
Smoothness	0.002	1	0.002	0.65	.4191	0.0003
Motion	0.005	1	0.005	2.15	.1425	0.0009
Shape	2.866	2	1.433	602.34	<.001	0.3298
Lighting × Proportion	0.006	1	0.006	2.62	.1059	0.0011
Lighting × Blurring	0.054	2	0.027	11.37	<.001	0.0092
Lighting × Smoothness	0.004	1	0.004	1.81	.1783	0.0007
Lighting × Motion	0.014	1	0.014	5.92	.0151	0.0024
Lighting × Shape	0.058	2	0.029	12.14	<.001	0.0098
Proportion × Blurring	0.016	2	0.008	3.33	.0361	0.0027
Proportion × Smoothness	<0.001	1	<0.001	0.03	.8552	<0.0001
Proportion × Motion	0.004	1	0.004	1.60	.2067	0.0007
Proportion × Shape	0.002	2	0.001	0.42	.6542	0.0003
Blurring × Smoothness	0.013	2	0.007	2.80	.0611	0.0023
Blurring × Motion	0.013	2	0.007	2.80	.0613	0.0023
Blurring × Shape	0.167	4	0.042	17.50	<.001	0.0277
Smoothness × Motion	0.002	1	0.002	0.79	.3750	0.0003
Smoothness × Shape	0.006	2	0.003	1.32	.2671	0.0011
Motion × Shape	0.032	2	0.016	6.76	.0012	0.0055
Lighting × Proportion × Motion	0.012	1	0.012	5.09	.0242	0.0021
Lighting × Blurring × Smoothness	0.021	2	0.010	4.34	.0131	0.0035
Lighting × Blurring × Shape	0.148	4	0.037	15.58	<.001	0.0248
Lighting × Smoothness × Shape	0.027	2	0.013	5.65	.0036	0.0046
Lighting × Motion × Shape	0.032	2	0.016	6.65	.0013	0.0054
Blurring × Motion × Shape	0.029	4	0.007	3.06	.0158	0.0050
Lighting × Blurring × Smoothness × Shape	0.046	4	0.012	4.86	<.001	0.0079
Error	5.824	2,448	0.002			
Total	11.859	2,591				

As a measure for the effect size, the last column shows the partial eta-squared (*η_p_*^2^) for each effect. Note that for higher order interaction effects, only results with *p* < .05 are shown.

## Appendix C—ANOVA Results for the Decision Time Data

**Table C1.** Results of the Six-Way Analysis of Variance of the Decision Time Data, Using All Six Experimental Factors: Lighting Condition (“Lighting”), Spatial Smoothness Proportion (“Proportion”), Edge Blurring (“Blurring”), Base Smoothness (“Smoothness”), Type of Surface Motion (“Motion”), and Bumpiness of the Surface’s Shape (“Shape”).

Source	Sum of squares	*df*	Mean squares	*F*	*p*	*η_p_* ^2^
Lighting	41.543	1	41.543	27.08	<.001	0.0109
Proportion	0.920	1	0.920	0.60	.4388	0.0002
Blurring	26.475	2	13.238	8.63	<.001	0.0070
Smoothness	49.078	1	49.078	31.99	<.001	0.0129
Motion	190.341	1	190.341	124.06	<.001	0.0482
Shape	182.182	2	182.182	59.37	<.001	0.0463
Lighting × Proportion	0.429	1	0.429	0.28	.5969	0.0001
Lighting × Blurring	5.642	2	2.821	1.84	.1592	0.0015
Lighting × Smoothness	0.009	1	0.009	0.01	.9393	<0.0001
Lighting × Motion	3.110	1	3.110	2.03	.1547	0.0008
Lighting × Shape	7.177	2	3.589	2.34	.0966	0.0019
Proportion × Blurring	0.692	2	0.346	0.23	.7981	0.0002
Proportion × Smoothness	1.224	1	1.224	0.80	.3718	0.0003
Proportion × Motion	0.048	1	0.048	0.03	.8595	<0.0001
Proportion × Shape	5.210	2	2.605	1.70	.1833	0.0014
Blurring × Smoothness	2.327	2	1.163	0.76	.4686	0.0006
Blurring × Motion	4.042	2	2.021	1.32	.2681	0.0011
Blurring × Shape	68.249	4	17.062	11.12	<.001	0.0178
Smoothness × Motion	0.804	1	0.804	0.52	.4692	0.0002
Smoothness × Shape	6.094	2	3.047	1.99	.1374	0.0016
Motion × Shape	13.767	2	6.884	4.49	.0113	0.0037
Lighting × Proportion × Blurring	13.012	2	6.506	4.24	.0145	0.0035
Blurring × Smoothness × Motion	11.101	2	5.550	3.62	.0270	0.0029
Lighting × Proportion × Blurring × Shape	20.170	4	5.043	3.29	.0107	0.0053
Lighting × Proportion × Motion × Shape	14.671	2	7.335	4.78	.0085	0.0039
Lighting × Blurring × Motion × Shape	19.824	4	4.956	3.23	.0118	0.0052
Proportion × Blurring × Smoothness × Shape	19.700	4	4.925	3.21	.0122	0.0052
Proportion × Blurring × Motion × Shape	23.733	4	5.933	3.87	.0039	0.0063
Error	3,755.79	2,448	1.534			
Total	4,638.54	2,591				

As a measure for the effect size, the last column shows the partial eta-squared (*η_p_*^2^) for each effect. Note that for higher order interaction effects, only results with *p* < .05 are shown.
